# Manufacture of Immunoglobulin Products for Patients with Primary Antibody Deficiencies – The Effect of Processing Conditions on Product Safety and Efficacy

**DOI:** 10.3389/fimmu.2014.00665

**Published:** 2014-12-23

**Authors:** Albert Farrugia, Isabella Quinti

**Affiliations:** ^1^Faculty of Medicine and Surgery, Department of Surgery, Centre for Orthopaedic Research, University of Western Australia, Perth, WA, Australia; ^2^College of Medicine, Biology and Environment, Australian National University, Acton, ACT, Australia; ^3^Plasma Protein Therapeutics Association, Annapolis, MD, USA; ^4^Department of Molecular Medicine, Sapienza University of Rome, Rome, Italy

**Keywords:** immunoglobulins, safety, manufacturing technology, hemolysis, thrombosis

## Abstract

Early preparations of immunoglobulin (Ig) manufactured from human plasma by ethanol (Cohn) fractionation were limited in their usefulness for substitution therapy in patients with primary antibody deficiencies (PAD), as Ig aggregates formed during manufacture resulted in severe systemic reactions in patients when given intravenously. Developments in manufacturing technology obviated this problem through the capacity to produce concentrated solutions of intact monomeric Ig, revolutionizing PAD treatment and improving patient life expectancy and quality of life. As the need for Ig has grown, manufacturers have refined further manufacturing technologies to improve yield from plasma and produce therapies, which are easier and less expensive to deliver. This has led to the substitution, partly or wholly, of ethanol precipitation by other techniques such as chromatography, and has also stimulated the production of highly concentrated solutions capable of rapid infusion. Ig products have been associated, since their inception, with certain adverse events, including infectious disease transmission, hemolysis, and thromboembolism. The introduction of standardized manufacturing processes and dedicated pathogen elimination steps has removed the risk of infectious disease, and the focus of attention has shifted to other problems, which appear to have increased over the past 5 years. These include hemolysis and thromboembolism, both the cause for substantial concern and the subject of recent regulatory scrutiny and actions. We review the development of manufacturing technology and the emerging evidence that changes for the optimization of yield and convenience has contributed to the recent incidents in certain adverse events. Industry measures under development will be discussed in terms of their potential to improve safety and optimize care for patients with PAD.

## Introduction

### Development of manufacturing methods for therapeutic immunoglobulin preparations

Immunoglobulins (Igs) may claim to be, historically, the first therapeutic plasma product, with Emil von Behring’s work on diphtheria antitoxin and von Behring and Kitasato’s demonstration (1) that serum of rabbits immunized with tetanus toxin contained activity against experimentally induced tetanus poison, which provided protection to non-immunized rabbits exposed to tetanus. For this work, von Behring was awarded the first Nobel Prize in Medicine or Physiology in 1901. Ehrlich (2) demonstrated that protection was correlated with the amount of antitoxin administered. Antibody preparations such as these, where protective antitoxin is generated through the immunization of animals, still have a role in the treatment of some conditions (3). Karelitz’s work showing that protection against measles was localized to the globulin portion of serum (4) and Tiselius’ characterization of serum proteins using electrophoresis (5) pointed to the role of gamma globulin in passive immunity. However, the manufacture of Ig solutions from a human source had to await Cohn’s development of methods to separate plasma fractions on a large scale (6), using ethanol as a precipitating agent in a series of separations manipulating pH, ionic strength, and temperature. Initial clinical experience with the immune serum globulin (ISG) fraction from Cohn’s scheme quickly led to limiting administration to the intramuscular and subcutaneous routes, as severe systemic reactions occurred in patients given this product intravenously.

Initially, ISG manufactured with Cohn’s method was limited to prophylaxis of certain microbial diseases, including polymyelitis, measles, mumps, pertussis, and hepatitis A (7). These preparations became redundant with the development of vaccines for the respective diseases. In 1952, Bruton (8) infused a child with undetectable “gamma globulin” levels and who suffered from chronic infections. Subcutaneous injections of ISG produced measurable gamma globulin levels and completely eliminated pneumococcal infections. Over the 1950s, human ISG became the standard treatment for patients with primary antibody deficiencies (PAD) who develop chronic bacterial infections (9). Preparations in which antibodies were enriched approximately 10- to 20-fold in 15–18% solutions were administered intramuscularly, a route, which caused problems. The intramuscular injection was painful, maximum serum levels were not reached before 24 h and could take several days, and *in vivo* recovery was usually less than 50% (10). At higher dosages, the preservative containing mercury caused increased concern (11). Intravenous administration would clearly obviate many of these problems but led to severe systemic reactions in 15–25% of patients. Patients with antibody deficiencies were particularly susceptible (12). The hypothesis that Ig aggregates in the preparations were leading to systemic complement activation (13) led manufacturers to explore ways of removing such aggregates as a way of preparing an intravenously administered product. These included digestion with enzymes such as pepsin and plasmin, leading to Ig fragments, which could bind antigens and were tolerated intravenously but which were lacking in effector functions and had very short intravascular lives. Further modifications involving chemical manipulation with β-Propiolactone, sulfonation, and alkylation resulted in intact molecules, with loss or modification of certain functions (14). By the end of the 1970s, various modifications of the original Cohn procedure resulted in a number of products containing >99% intact, monomeric Ig, well-tolerated intravenously, and able to be infused in high volumes and result in a prolonged presence of high Ig levels in the patient’s blood. Coupled with the increasing range of indications for Ig in a number of autoimmune and inflammatory diseases, this ability to delivery higher dosages, and improve clinical outcomes, in immune deficient patients ushered in the current era of ever increasing usages of immune globulin therapies.

## Immunoglobulin Therapies – Current Products, Current Issues

Bruton first treated agammaglobulinemia in 1952 with ISG administered subcutaneously (8), a route, which was supplanted first by intramuscular and, since the 1980s, intravenous, products. Over the past decade, subcutaneous products have been developed with an enhanced capacity to allow home care and avoid adverse events (15). More recently, most manufacturers have acquired approval to market products for both intravenous and subcutaneous routes with increased strengths, with Ig concentrations of up to 20% compared to the mainstream intravenous products of 4–5%, as well as faster infusion rates. Most of these developments have been spurred by economic considerations, aimed at minimizing time in hospitals (16), while allowing patients more freedom through home therapy. In addition, reports that increasing dosages result in a continuing improvement in clinical outcomes in PID (17) have possibly encouraged the development of more concentrated solutions. As Ig consumption has continued to increase, payer influence in therapeutic practice, particularly in USA, has become a matter of concern (18, 19). Increasingly, the techniques of mainstream pharmacoeconomics have been used to question the allocation of health care resources to Ig, although such analyses have been limited to indications other than PID so far (20, 21).

## Historically Recognized Adverse Effects of Ig Therapies

Ig therapies have been associated with a number of adverse side effects, which have been extensively reviewed in Ref. (22). The present work does not intend to reiterate these efforts, which have all drawn attention to the rarity of serious side effects. Rather, we intend to focus on effects, which, at one time or another, increased in frequency as a result of what were, retrospectively, recognized as ensuing from changes in plasma collection and manufacturing methods. We have chosen three such adverse events: pathogen transmission, thromboembolism, and hemolysis. Our aim is to demonstrate how the uniqueness of each manufacturer’s process influences the product, and reinforces the concept that Ig therapies, as biological drugs, cannot be viewed as generic and interchangeable.

## Pathogen Transmission Issues in Ig Therapies

All plasma protein therapies had a history of transmitting pathogens before the current era characterized by robust pathogen elimination steps in the manufacture came into effect. Intramuscular Ig products transmitted hepatitis B on rare occasions (23) but were not associated with the transmission of other blood-borne viruses. The presence of antibodies to the respective viruses, which was the basis of the protective action of specific IG products, was assumed to account for the rarity of transmission by ISG. In addition, the sequential precipitation of fractions during the Cohn process was thought to partition virus away from the therapeutic Ig fraction (24). Initial experience with early intravenous preparations seemed to support this safety record.

Over the early 1980s, several IVIG preparations manufactured at pilot scale transmitted Non A-Non B hepatitis, subsequently shown to be hepatitis C (HCV). Changes in the finishing steps used to remove ethanol from the final product were initially ascribed as the cause of the different infectivity of these IV preparations compared to their IM counterparts (25), as the Cohn system common to both products was considered to have a high HCV clearing capacity (26). This was subsequently shown to be modest in the absence of a specific virus inactivating step (27). Such steps were introduced into the manufacture of IVIG from the early 1990s onward, but not in time to prevent two major outbreaks of HCV in recipients of intravenous Ig.

## HCV from Anti-D – “*The Road to Hell*…

In 1994, reports emerged of HCV transmission in Irish women, subsequently linked to the administration of anti-D Ig (28). Historically, anti-D Ig is an intramuscular preparation, which, up to the time of this incident, had a strong safety record. Investigations showed that the transmissions occurred during 1977 and 1978, following administration of an anti-D product manufactured on a small scale in the Irish blood service’s facility in Dublin. The method used was a chromatographic technique developed in East Germany some years before (29), and, as in the case of the parent facility, was introduced in order to maximize the yield of anti-D from domestic plasma, and achieve self-sufficiency in this product in the absence of a domestic Cohn fractionation facility (30). Concurrently, HCV from the original product was also shown to transmit HCV (31). Genomic studies revealed concordance between the HCV in the product and that in the infected patients, and the original contamination was traced to one infected donor contributing through plasmapheresis for therapeutic purposes (32).

This epidemic in Irish women affected some 400 recipients of a product, which has, in its classical form, had an unblemished safety record (33). A process executed by the Irish government as a result of this catastrophe led to profound reform in the blood service and its oversight (34). The Irish Blood Service, which was relatively unsupervised at the time of these events, chose, in its quest for self-sufficiency in anti-D, a manufacturing method, which was relatively new and untested through the long years of experience, which had resulted in high levels of confidence in Cohn fractionated product. Clearly, the chromatographic method had little capacity to eliminate the viral load resulting from one repeat plasmapheresis donor. Reliance on the safety history of anti-D ignored the effect of a total change in manufacture, in a modest facility with little adherence to the principles of good manufacturing practice, and where high-risk donors were accepted for contribution to the plasma pool. In the sad history of pathogen transmission through plasma products, it is difficult to find another accident of such obvious culpability. It also provides a salutary example of the difficulty in predicting the outcome of changes in established manufacturing techniques on the safety of products. It is not the only such example.

## The Gammagard Incident – ………is Paved with Good Intentions”

The Irish/German incident involved an intravenous product administered to healthy women. In 1994, reports emerged indicating an epidemic of HCV in recipients of polyclonal IVIG from a specific manufacturer. This product – Gammagard from Baxter Healthcare Corporation – had been licensed in USA since 1986 and had demonstrated a good safety record, including lack of evidence of transmission of Non A, Non B hepatitis (35). Despite this, an apparent epidemic of HCV occurred in recipients given Gammagard over 1993–94 (36). Gammagard – and all other plasma products – differed in one important feature in 1993 relative to 1986; in the intervening period, the characterization of HCV and the introduction of rapidly succeeding tests for anti-HCV antibody resulted in the rapid diminishment of anti-HCV from transfusion products and the raw material for plasma fractionation. While this immediately resulted in a substantial increase in the safety of transfusion products, a concurrent increase in the safety of pooled plasma products could not be assured. The safety of plasma products is primarily assured through pathogen elimination procedures, which enhance safety margins to a much higher extent than the donor selection and testing measures, which still underpin the safety of single or small pool transfusion therapies (37).

Scientific opinion at the time of the introduction of the anti-HCV test included those who speculated that the exclusion of anti-HCV antibodies from fractionation pools might decrease product safety (38). The US FDA did not require HCV antibody testing for source plasma for fractionation until experiments in their laboratories indicated that the removal of such antibodies did not affect safety from HCV (39). These experiments used plasma tested negative with the first-generation anti-HCV test, which does not exclude all antibody units, particularly units with HCV antibody specificities against HCV envelope proteins (40). Other works showed that the Cohn system did not clear HCV from the final therapeutic fraction (26). After investigating the Gammagard incident (41), the FDA scientists hypothesized that the exclusion of anti-HCV antibodies against HCV envelope proteins through the use of second and third generation anti-HCV tests had affected the portioning of HCV during fractionation, to result in free, viable virus being deposited in the Ig fraction, which otherwise would have been neutralized by antibodies to HCV (42, 43). Although the data available make this hypothesis persuasive, it is regrettable that a specific viral inactivation step, which had been under development for this product for some years had not been introduced in time to obviate any viral transmission. The step was hastily approved by the FDA and introduced in the wake of the Gammagard incident (36).

This incident continues to accentuate the point made above that the safety of Ig products cannot be assumed on the basis of historical experience, when any variation in the complex nexus of processes, which constitute the manufacture of Ig products is instituted. In this particular instance, a test, which unquestionably led to the enhancement of the safety of transfusion products was insufficiently assessed in its potential role in Ig safety, and was assumed to contribute to safety in the absence of specific viral safety measures. The rarity of viral transmission from Ig products was considered to be a good foundation for variations in their manufacture, in the absence of any scientific validation. The presumption of linearity in blood safety, i.e., assuming a capacity for predicting the future safety of plasma products on the basis of past experience, was fulfilled following these incidents through the efforts of the plasma industry and its regulatory overseers in introducing the complex nexus of measures for ensuring the safety of donors through the exclusion of high-risk groups, the screening of donations and plasma pools with nucleic acid tests to minimize the inclusion of donors in the silent “window period” of infection (44) and the implementation of dedicated pathogen reduction steps in the manufacture, which ensured that Ig therapies were safe from established and emerging pathogens. In particular, the role of pathogen reduction steps in overcoming the “Non-Linearity” of blood safety risks cannot be over-emphasized (45). Early precautionistic measures implemented by regulators to minimize exposure to products potentially contaminated with prions from patients with transmissible spongiform encephalopathies (TSEs) led to product recalls and shortages (46). In the absence of a screening test, these measures could only be revised with the introduction of steps in the manufacture, which were shown to decrease prion contamination during plasma fractionation (47). The absence of infection in a patient who received Ig manufactured from a donor who subsequently developed variant Creutzfeldt–Jakob disease (vCJD) demonstrates the effectiveness of these measures (48).

The salutary experiences encountered during the evolution of Ig therapies to their current status of safety from blood borne pathogens, unfortunately, did not prevent similar problems in other areas as a result of manufacturing changes in succeeding years, as we shall now review.

## Thromboembolic Events

Until recently, thromboembolic sequelae (TEEs) of Ig administration were relatively rarely reported, although their consequences were severe. With the increasing level of adverse event monitoring required of plasma therapies, increasing reports of these events have been published. Given the dramatic and often life threatening nature of these incidents, the increase in reports may be assumed to reflect an increased incidence. The interest and concern in this issue increased considerably when a major product was recalled from the North American and European markets because of an increased incidence in thromboembolic events. The product – Octagam from Octapharma – had a good safety record prior to this incident (49). The regulatory agencies and the industry collaborated in investigating the putative causes in the increased incidence of TEEs, including the Octagam problem, with the US FDA holding a workshop in 2011 whose proceedings are available (50). As a result of these investigations, the causative agent involved in the increased thrombogenicity of Ig preparations was determined to be coagulation Factor XI and XIa, generated in increased amounts following changes in the manufacturing procedures introduced over the previous years. The regulatory agencies mandated the introduction of changes in the manufacturing schemes of products determined to have increased Factor XI (51), and provided guidance and reference preparations for testing methods for FXIa in Ig (52). Further guidance was offered subsequently emphasizing the need to factor in patient characteristics in the administration of Ig (53), following epidemiological investigation, which showed that the majority of affected patients had pro-thrombotic co-morbidities (54). A high level warning on the risk of thrombosis was mandated for the product information available to patients and prescribers (55).

These regulatory measures indicated a somewhat belated recognition of the multifactorial nature of the problem of TEEs from Ig therapies. It appears definite that a previously very rare adverse event increased in reported incidence in the early 2000s. The attribution of increased FXIa levels in the product as a causal agent precedes this period (56), as do many of the manufacturing methods and variations described for the products associated with the highest TEE incidence (57, 58). These products included Octagam (Octapharma) and Vivaglobin (CSL), a 5% (for the formulation associated with TEE) intravenous and a 16% subcutaneous preparation, respectively. The variations in the methods, which are referred to in the literature describing the TEE incidents are common measures used by most manufacturers, and must be presumed, if they were introduced following initial market approval, to have been incorporated into standard practice following regulatory scrutiny and clinical studies. Any such regulatory processes failed to detect any problems, providing a sober example of the limitations of mainstream regulation and the difference between efficacy as assessed by regulators and effectiveness as reflected from the real world of therapeutic practice (59). Recalls of product only occurred for Octagam, despite an FDA investigation in which interrogation of a large US health insurance claims revealed higher risks with Vivaglobin (60). These risks were also higher in European countries but not to the levels reported in USA (61). Vivaglobin was subjected to scrutiny and warnings (62), and was withdrawn from the market concurrently with these events to be replaced with a 20% SCIG – Hizentra (63) – using a process validated to exclude pro-coagulants (64). The paucity of TEE reports following these incidents indicates that the measures introduced led to the desired effects. It would heighten the understanding of this and similar problems if investigations were to be done and reported on any changes in clinical and product administrative practices, such as infusion rates. The continuing increase in consumption of Ig therapies (65) does not indicate any modification in dosage and other issues, which are considered to play a role in TEEs (66).

## Hemolysis

Like TEE, intravascular hemolysis following Ig administration is a rare adverse event, which seems to be increasing in frequency (67, 68). A workshop was again convened by the FDA in January 2014 to assess this problem. Transcripts are available (69). Regulatory laboratories have determined that products with high concentrations of Ig, which are produced by revised versions of the classical fractionation scheme are preferentially associated with reactions (70) and are also associated with the highest titers of blood group antibodies anti-A and anti-B (71). One company obtained regulatory approval for updates to its safety information regarding hemolysis, specifying the role of dosage and the relationship to patient blood group and underlying disease state (72).

Investigations from the industry indicate that the apparent increase in the incidence of hemolysis may be related, as with TEEs, to changes in manufacturing methods introduced over recent years in order to increase the yield of Ig from plasma. Classical Cohn fractionation has been shown to result in a substantial reduction in isoagglutinin titer through the sequential removal through precipitation of Fraction I and, in particular, Fraction III (73, 74), and modifications of the scheme to omit these sequential precipitations or to purify Ig with different precipitating/chromatographic techniques result in products with little removal of isoagglutinins. As a result, manufacturers are assessing the feasibility of introducing further modifications in the fractionation of the current generation of products in order to remove isoagglutinins (75), as well as screening donors to exclude high isoagglutinin titer donations from the fractionation pool (76).

## Reflections on Recent Adverse Event Reports

The past 20 years have seen the following developments in Ig therapies:

1. The introduction, in common with all plasma protein therapies, of measures to decrease pathogen transmission.
2. A widening of indications, in particular to treat autoimmune neuropathies such as Chronic Inflammatory Demyelinating Polyneuropathy (CIDP), GBS, and MMN.
3. A consequent drive to increase Ig yield from plasma, through modification of the classic ethanol precipitation scheme or its abandonment to other techniques.
4. A consequent increase in the cost of treating these disorders, leading to scrutiny by reimbursement agencies and pressure to cut costs.
5. The introduction of more concentrated solutions administered intravenously through high infusion rates, and subcutaneously, at least partly fueled by the need to decrease costs through lessening hospital length of stay periods.
6. The emergence of adverse events at higher incidence than historically expected.

The contention of this work is that developments 1–5 led to development 6. We propose this as a “Popperian” hypothesis, able to be falsified through evidence. We do not assert this with the aim of ascribing blame, but rather as a route to a better understanding of Ig therapies and the need to address their benefits as well as their limitations. We propose the following aspects merit consideration.

## The Difficulty in Predicting Outcomes in Biological Therapeutics

Murphy has pointed out that a linear approach to predict the safety of blood-derived therapies is not desirable (77), proposing that many features of the blood system show characteristics of the chaotic behavior found in systems composed of complex interdependent components. Reflecting on the myriad of factors, which may affect the safety and efficacy of a product made, as are Ig therapies, from the blood of tens of thousands of individuals using a complex manufacturing process, the capacity for apparently minor and well-intentioned variations in donor composition and technology to result in deleterious outcomes is not to be wondered at. Hence, oft-stated claims about “zero risk” plasma therapies and “well-controlled” manufacturing processes risk being hubristic at best. Considering the scenarios, which ultimately led to pathogen transmission from blood in the past 20 years resonate, superficially, more to science fiction than to what was current empirical knowledge (78), any hypothesis proposing caution before the implementation of any potential perturbations in the blood safety landscape deserves to be treated with respect. Hence, the doubts shown on the advisability of anti-HCV testing for plasma destined for Ig production were shown to be justified, and the empirical evidence supposedly disproving this hypothesis was shown to be incomplete, unfortunately after affected product transmitted HCV. Similarly, changes in the manufacturing method intended to increase yield and lead to “self-sufficiency” resulted in infections in healthy women given anti-D, a product with an unblemished safety record when manufactured in the classical fractionation scheme. Clearly, improving blood transfusion safety through HCV antibody testing and improving anti-D access from a domestic source were well-intentioned policies, which, however, failed to take into account the specialized and complex area of plasma protein manufacture, with its vulnerability to processing changes. Similarly, the more recent events whereby adverse events hitherto rare in incidence became more common following, again, manufacturing changes whose potential effects on the product were insufficiently considered to demonstrate a failure by industry and regulators alike, in not appreciating that the rarity of such events with the classical products indicated a robustness in the manufacturing method, long derided as “bucket chemistry,” which the more recent technologies failed to attain. When Oncley and co-workers had specifically reported in 1949 that Fraction III in the Cohn system contained the bulk of the isoagglutinins (79), was it to be wondered when omission of this step led to hemolytic sequelae in patients? We would contend that the evidence for safety of Ig therapies, before these changes and the subsequent problems, was evident, and “If it ain’t broke, don’t fix it” might have played a role in the decision-making process.

## The Need for Higher Yields

As we have discussed, the driver for changes in the classical technologies has been the need for higher yields of Ig. Ig yield from the classical Cohn scheme is of the order of 3 g/L of plasma at best (14). The current generation of products using modified techniques is obtained at yields of 3.5–5.4 g/L (14, 80), compared to plasma levels of around 6.5–8.5 g/L in most of the raw material used by the manufacturers (81). We presume that further enhancements in yield are possible and that manufacturers will continue to examine the feasibility of modifications to the fractionation process. Such modifications generally require regulatory approval, which may include evidence of clinical efficacy and safety. The failure of agencies across the world to specify regulatory processes able to detect the harmful features introduced in previous modifications such as those discussed in this work does not augur well for the safety of any further changes.

## Clinical Need of Ig Therapies

An analysis of the legitimate clinical need for Ig is legitimate in order to assess the demand for plasma and ensure optimal care. An analysis of the mainstay indication – substitution therapy in patients with humoral immune deficiencies – using published evidence for the variables currently influencing clinical need determined a requirement of 72 g per thousand population for this purpose (82), a need, which exceeds the actual current usage in most countries. This indication represents around 30% of overall Ig usage in published sources (83). Around 60% of Ig is used for immunosuppressive treatment of a number of autoimmune disorders, with particularly large volumes consumed by neuropathies such as CIDP. Although much is made about the so-called “off-label” indications of Ig, there is little doubt that, in the established markets, the vast majority of prescriptions for Ig are for evidence-based indications. This is indicated by the outcomes in the Australian system, where delivery of Ig, through a single government payer, is conditional to adherence to a set of prescribing guidelines, which allocate the product strictly and solely to indications with the highest level of evidence (84). It is, therefore, likely that, as diagnosis and therapeutic practice improve, the demand for Ig will continue the inexorable rise, which has been recorded over the past 10 years.

It behooves all players in this area to scrutinize such practice. The increase in Ig consumption is based on the ability to deliver large doses of product through the intravenous route and, increasingly, through subcutaneously administered deposits. More understanding is needed on the optimal dosage regimen for the different patient groups. In immune deficiency, one hypothesis, fueled by a meta-analysis of clinical trials, proposes that continuous increases of Ig dosage to higher through levels will continue to lead to clinical benefit through decreasing pneumonia episodes (17). This hypothesis suggests that patients should attain Ig through levels to at least the mid-normal range of Ig levels. In contrast, the work of Quinti et al. (85) on a prospectively studied PID patient cohort found no benefit in the same clinical indicator when through levels were above 400 mg/dL. We suggest that, in an era of advocacy for personalized medicine (86), focusing on the clinical condition of patients and individualizing therapy according to such data needs to replace the “guideline” driven practices, which have underpinned much of therapeutics, including the treatment of patients with Ig therapies. Milito et al. (87), using a pharmacokinetic-driven approach, have shown that stratifying PID patients according to clinical phenotype permits the individualization of Ig dosage and results in significantly lower total Ig usage than the standard dosage regimen. While large prospective multicenter studies are needed to confirm and augment this work, we suggest that agencies seeking to ensure, for financial reasons, more “rational” Ig therapy would do well to consider the funding of such studies if patient care is to be optimized.

The considerable Ig usage, in doses far exceeding those used in PID, for autoimmune disorders also requires review and more investigation. The ways in which Ig modulates the immune system in these diseases is still the subject of research and is probably multifactorial, but evidence for the role of the Fc portion of Ig appears compelling (88). Enhancing the sialylation of the Fc portion has been proposed as a specific route to more targeted Ig therapies for autoimmune and inflammatory states, allowing more potent preparations at lower dosages of total Ig (89), and a commercial development, currently lacking in detail, has been announced (90). Keeping in mind that the majority of the recently encountered adverse events have affected patients with these disorders, and that causality has been ascribed to high doses, further work on the establishment and development of this therapy is a priority and a challenge for industry and regulators alike.

## Summary and Conclusion

Ig therapies are life-saving medicines, which have revolutionized the treatment of a large number of rare and severe chronic immune deficiency and autoimmune disorders. Industry investment in the improvement and administration of these therapies has increased greatly the number of patients, which can benefit, enhancing life expectancy and quality of life. This has also led to an increase in the adverse events associated with these medicines. Current regulatory constructs are unable to predict such events prior to the introduction of the therapies on the market place. Hence, an effort to better understand the manufacture and indications of these therapies is mandatory. Rather than treating Ig as a form of “gasoline,” putting more in the patient “tank” in order to get more clinical “mileage,” personalizing patient care and developing a new generation of more specific, targeted therapies should be the focus of the coming years of therapeutics. We suggest that the seeds of such approaches are appearing, and that more studies will develop these if sufficiently resourced. We propose that the current era of financial stringency should not be the excuse for rationing Ig therapies to the detriment of patient care, but should act as the spur to improving our understanding and use of these crucial natural molecules.

## Conflict of Interest Statement

Albert Farrugia provides paid consultancy services to the manufacturers of immunoglobulin therapies.

## References

[1] von Behring EA Kitasato S U ber das zustandekommen der diphtherie-immunitat und der tetanus-immunitat bei tieren. Dtsch Med Wochenschr (1890) 16:1113–4 10.1055/s-0029-1207589

[2] Ehrlich P Experimentelle untersuchungen uber immunita t. I. Uber Ricin. II. Uber Abrin. Dtsch Med Wochenschr (1891) 17(976–9):1218–9 10.1055/s-0029-1206825

[3] Stiehm ER Appropriate therapeutic use of immunoglobulin. Transfus Med Rev (1996) 10:203–21 10.1016/S0887-7963(96)80060-5 8809970

[4] Karelitz S Prophylaxis against measles with the globulin fraction of immune adult serum. Am J Dis Child (1938) 55:768–75.

[5] Tiselius A Kabat EA Electrophoresis of immune serum. Science (1938) 87:416–7 10.1126/science.87.2262.416-a 17829941

[6] Cohn EJ Luetscher JA Jr Oncley JL Armstrong SH Jr Davis BD Preparation and properties of serum and plasma proteins. J Am Chem Soc (1940) 62:3396–400 10.1021/ja01869a032

[7] Barandun S Immunoglobulins: history, present trends and safety aspects. In: Waters AH Webster ADB, editors. Intravenous Immunoglobulins, Royal Society of Medicine International Conference and Symposium Series No. 84 London: Royal Society of Medicine (1985). p. 3–10.

[8] Bruton OC Agammaglobulinema. Pediatrics (1952) 9:722–7.14929630

[9] Janeway CA Rosen FS The gamma globulins. IV. Therapeutic uses of gamma globulin. N Engl J Med (1966) 275:826–31 10.1056/NEJM196610132751508 4161939

[10] Smith GN Griffiths B Mollison D Mollison PL Uptake of IgG after intramuscular and subcutaneous injection. Lancet (1972) 1(7762):1208–12 10.1016/S0140-6736(72)90926-9 4113191

[11] Haeney MR Carter GF Yeoman WB Thompson RA. Long-term parenteral exposure to mercury in patients with hypogammaglobulinemia. BMJ (1979) 2:12–4. 10.1136/bmj.2.6181.12 466248 PMC1595823

[12] Eibl MM History of immunoglobulin replacement. Immunol Allergy Clin North Am (2008) 28(4):737–64 10.1016/j.iac.2008.06.004 18940572

[13] Barandun S Kistler P Jeunet F Isliker H Intravenous administration of human gamma globulin. Vox Sang (1962) 7:157–74 10.1111/j.1423-0410.1962.tb03240.x 13864762

[14] Radosevich M Burnouf T. Intravenous immunoglobulin G: trends in production methods, quality control and quality assurance. Vox Sang (2010) 98(1):12–28. 10.1111/j.1423-0410.2009.01226.x 19660029

[15] Wasserman RL. Progress in gammaglobulin therapy for immunodeficiency: from subcutaneous to intravenous infusions and back again. J Clin Immunol (2012) 32(6):1153–64. 10.1007/s10875-012-9740-x 22828788

[16] Gerth WC Betschel SD Zbrozek AS. Implications to payers of switch from hospital-based intravenous immunoglobulin to home-based subcutaneous immunoglobulin therapy in patients with primary and secondary immunodeficiencies in Canada. Allergy Asthma Clin Immunol (2014) 10(1):23. 10.1186/1710-1492-10-23 24872821 PMC4036390

[17] Orange JS Grossman WJ Navickis RJ Wilkes MM. Impact of trough IgG on pneumonia incidence in primary immunodeficiency: a meta-analysis of clinical studies. Clin Immunol (2010) 137(1):21–30. 10.1016/j.clim.2010.06.012 20675197

[18] Shapiro RS Boyle M. Payor issues: barriers to optimal management of patients with primary immunodeficiency. J Clin Immunol (2012) 32(Suppl 2):S410–4. 10.1007/s10875-012-9759-z 22918575

[19] Bonagura VR Marchlewski R Cox A Rosenthal DW Biologic IgG level in primary immunodeficiency disease: the IgG level that protects against recurrent infection. J Allergy Clin Immunol (2008) 122(1):210–2 10.1016/j.jaci.2008.04.044 18602574

[20] Blackhouse G Gaebel K Xie F Campbell K Assasi N Tarride JE Cost-utility of Intravenous Immunoglobulin (IVIG) compared with corticosteroids for the treatment of chronic inflammatory demyelinating polyneuropathy (CIDP) in Canada. Cost Eff Resour Alloc (2010) 17(8):14. 10.1186/1478-7547-8-14 20565778 PMC2903512

[21] Liu Z Albon E Hyde C The Effectiveness and Cost Effectiveness of Immunoglobulin Replacement Therapy for Primary Immunodeficiency and Chronic Lymphocytic Leukaemia: A Systematic Review and Economic Evaluation. (2014). Available from: http://www.birmingham.ac.uk/Documents/college-mds/haps/projects/WMHTAC/REPreports/2005/IgRT.pdf

[22] Nydegger UE Sturzenegger M Adverse effects of intravenous immunoglobulin therapy. Drug Saf (1999) 21(3):171–85 10.2165/00002018-199921030-00003 10487396

[23] Tabor E Gerety RJ Transmission of hepatitis B by immuno serum globulin. Lancet (1979) 2(8155):1293 10.1016/S0140-6736(79)92296-7 93197

[24] Boschetti N Stucki M Späth PJ Kempf C. Virus safety of intravenous immunoglobulin: future challenges. Clin Rev Allergy Immunol (2005) 29(3):333–44. 10.1385/CRIAI:29:3:333 16391410 PMC7090396

[25] Yap PL. Intravenous immunoglobulin and hepatitis C virus: an overview of transmission episodes with emphasis on manufacturing data. Clin Ther (1996) 18(Suppl B):43–58. 10.1016/S0149-2918(96)80195-0 8930441

[26] Yei S Yu MW Tankersley DL. Partitioning of hepatitis C virus during Cohn-Oncley fractionation of plasma. Transfusion (1992) 32:824–8. 10.1046/j.1537-2995.1992.32993110753.x 1335184

[27] Scheiblauer H Nübling M Willkommen H Löwer J. Prevalence of hepatitis C virus in plasma pools and the effectiveness of cold ethanol fractionation. Clin Ther (1996) 18(Suppl B):59–70. 10.1016/S0149-2918(96)80196-2 8930442

[28] Power JP Lawlor E Davidson F Yap PL Kenny-Walsh E Whelton MJ Hepatitis C viraemia in recipients of Irish intravenous anti-D immunoglobulin. Lancet (1994) 334:116–7. 7934528 10.1016/s0140-6736(94)90679-3

[29] Hoppe HH Mester T Henning W Krebs HJ Prevention of Rh-immunisation. Modified production of IgG anti-Rh for intravenous application by ion-exchange chromatography (IEC). Vox Sang (1973) 25:308–16 10.1111/j.1423-0410.1973.tb04378.x 4201742

[30] Cunningham CJ Production of human immunoglobulin anti-D (Rh0) for intravenous administration, for a national Rh prophylaxis programme [proceedings]. Biochem Soc Trans (1980) 8(2):178–9. 6245973 10.1042/bst0080178

[31] Dittmann S Roggendorf M Dürkop J Wiese M Lorbeer B Deinhardt F. Long-term persistence of hepatitis C virus antibodies in a single source outbreak. J Hepatol (1991) 13:323–7. 10.1016/0168-8278(91)90076-N 1725527

[32] Power JP Lawlor E Davidson F Holmes EC Yap PL Simmonds P. Molecular epidemiology of an outbreak of infection with hepatitis C virus in recipients of anti-D immunoglobulin. Lancet (1995) 345(8959):1211–3. 10.1016/S0140-6736(95)91993-7 7739308

[33] Akehurst C Hepatitis C virus infection from contaminated anti-D immune globulin in Ireland. Euro Surveill (1999) 3(18):29.

[34] Finlay TA Report of the tribunal of inquiry into the blood transfusion service board. Irish Health Separtment (1997). Available from http://www.lenus.ie/hse/bitstream/10147/45442/1/7843.pdf

[35] Piszkiewicz D Kingdon H Lee ML Hooper J. The incidence of HTLV-III/LAV seroconversion and non-A, non-B hepatitis in recipients of plasma products. Dev Biol Stand (1987) 67:327–31. 2440745

[36] Gomperts ED. Gammagard and reported hepatitis C virus episodes. Clin Ther (1996) 18(Suppl B):3–8. 10.1016/S0149-2918(96)80192-5 8930438

[37] Kreil TR New Technologies for Virus Detection: Where Donor Screening is Going. Presentation to The 8th Global Forum of the World Federation of Hemophilia (2013). Available from: http://www1.wfh.org/docs/en/Events/GF2013/GF2013_Kreil.pdf

[38] Finlayson JS Tankersley DL Anti-HCV screening and plasma fractionation: the case against. Lancet (1990) 335:1274–5 10.1016/0140-6736(90)91335-8 1971337

[39] Biswas RM Nedjar S Wilson LT Mitchell FD Snoy PJ Finlayson JS The effect on the safety of intravenous immunoglobulin of testing plasma for antibody to hepatitis C. Transfusion (1994) 34(2):100–4. 10.1046/j.1537-2995.1994.34294143934.x 7508642

[40] Gretch DR Diagnostic tests for hepatitis C. Hepatology (1997) 26(3 Suppl 1):43S–7S 10.1002/hep.510260708 9305663

[41] Bresee JS Mast EE Coleman PJ Baron MJ Schonberger LB Alter MJ Hepatitis C virus infection associated with administration of intravenous immune globulin. A cohort study. JAMA (1996) 276(19):1563–7. 10.1001/jama.1996.03540190035026 8918853

[42] Yu MW Mason BL Guo ZP Tankersley DL Safety of intravenous immunoglobulin with regard to hepatitis C virus. Clin Ther (1996) 18(Suppl B):71–2 10.1016/S0149-2918(96)80197-4 8930443

[43] Yu MW Mason BL Guo ZP Renzi PM Tankersley DL Safety of immunoglobulins from HCV. In: Rizzetto M Purcell RH Gerin JC Verme G, editors. Viral Hepatitis and Liver Disease. Turin: Edizoni Minerva Medica (1997). p. 276–9.

[44] Food and Drug Administration. Guidance for Industry: Use of Nucleic Acid Tests on Pooled and Individual Samples from Donors of Whole Blood and Blood Components (Including Source Plasma and Source Leukocytes) to Adequately and Appropriately Reduce the Risk of Transmission of HIV-1 and HCV 2004. (2014). Available from: http://www.fda.gov/BiologicsBloodVaccines/GuidanceComplianceRegulatoryInformation/Guidances/Blood/ucm074934.htm

[45] Farrugia A Kreil T Chikungunya, Ebola and the crisis in the blood safety paradigm. Presented to the Annual Meeting of the AABB. Philadelphia, PA (2014). Available from: https://www.youtube.com/watch?v=MMZzhhDV-To

[46] US Government Accountability Office. Blood Safety: Recalls and Withdrawals of Plasma Products. (1998). Available from: http://www.gpo.gov/fdsys/pkg/GAOREPORTS-T-HEHS-98-166/html/GAOREPORTS-T-HEHS-98-166.htm

[47] Foster PR Welch AG McLean C Griffin BD Hardy JC Bartley A Studies on the removal of abnormal prion protein by processes used in the manufacture of human plasma products. Vox Sang (2000) 78(2):86–95. 10.1046/j.1423-0410.2000.7820086.x 10765143

[48] El-Shanawany T Jolles S Unsworth DJ Williams P A recipient of immunoglobulin from a donor who developed vCJD. Vox Sang (2009) 96(3):270 10.1111/j.1423-0410.2008.01148.x 19522885

[49] Debes A Bauer M Kremer S. Tolerability and safety of the intravenous immunoglobulin Octagam: a 10-year prospective observational study. Pharmacoepidemiol Drug Saf (2007) 16(9):1038–47. 10.1002/pds.1449 17636556

[50] US Food and Drug Administration. Risk Mitigation Strategies to Address Potential Procoagulant Activity in Immune Globulin Products Presentations (2014). Available from: http://www.fda.gov/BiologicsBloodVaccines/NewsEvents/WorkshopsMeetingsConferences/ucm247285.htm

[51] European Medicines Agency. European Medicines Agency Reminds Marketing-Authorisation Holders about Revised Monographs for Human normal immunoglobulin. (2012). Available from: http://www.ema.europa.eu/ema/index.jsp?curl=pages/news_and_events/news/2012/09/news_detail_001617.jsp&mid=WC0b01ac058004d5c1

[52] Gray E Wilmot H Hogwood J Rigsby P Evaluation of the proposed WHO 1st Reference Reagent for Activated Blood Coagulation Factor XI (FXIa), Human. Expert Committee On Biological Standardization. (2012). Available from: http://apps.who.int/iris/bitstream/10665/78047/1/WHO_BS_2012.2206_eng.pdf?ua=1

[53] Food and Drug Administration. FDA Safety Communication: Updated Information on the Risks of Thrombosis and Hemolysis Potentially Related to Administration of Intravenous, Subcutaneous and Intramuscular Human Immune Globulin Products. (2012). Available from: http://www.fda.gov/BiologicsBloodVaccines/SafetyAvailability/ucm327934.htm

[54] Jacobs K Analysis of Spontaneously Reported Thromboembolic Events (TEE) for Patients Under IVIG Treatment, 2005 – 2010. Presented to FDA Workshop on Risk Mitigation Strategies to Address Potential Procoagulant Activity in Immune Globulin Products. (2011). Available from: http://www.fda.gov/downloads/BiologicsBloodVaccines/NewsEvents/WorkshopsMeetingsConferences/UCM260783.pdf

[55] Food and Drug Administration. New Boxed Warning for Thrombosis Related to Human Immune Globulin Products. (2013). Available from: http://www.fda.gov/BiologicsBloodVaccines/SafetyAvailability/ucm375096.htm

[56] Wolberg AS Kon RH Monroe DM Hoffman M. Coagulation factor XI is a contaminant in intravenous immunoglobulin preparations. Am J Hematol (2000) 65(1):30–4. 10936860 10.1002/1096-8652(200009)65:1<30::aid-ajh5>3.0.co;2-j

[57] European Medicines Agency. Assessment Report for Octagam and Associated Names. (2011). Available from: http://www.ema.europa.eu/docs/en_GB/document_library/Referrals_document/Octagam_31/WC500154855.pdf

[58] European Medicines Agency. Referral Assessment Report for Vivaglobin and Associated Names (Human Normal Immunoglobulin for Injection – Subcutaneous Use). (2012). Available from: http://www.ema.europa.eu/docs/en_GB/document_library/Referrals_document/Vivaglobin_36/WC500154569.pdf

[59] Singal AG Higgins PD Waljee AK. A primer on effectiveness and efficacy trials. Clin Transl Gastroenterol (2014) 5:e45. 10.1038/ctg.2013.13 24384867 PMC3912314

[60] Sridhar G Ekezue BF Izurieta HS Selvam N Ovanesov MV Divan HA Immune globulins and same-day thrombotic events as recorded in a large health care database during 2008 to 2012. Transfusion (2014) 54(10):2553–65. 10.1111/trf.12663 24804899

[61] Funk MB Gross N Gross S Hunfeld A Lohmann A Guenay S Thromboembolic events associated with immunoglobulin treatment. Vox Sang (2013) 105(1):54–64 10.1111/vox.12025 23398249

[62] Food and Drug Administration. Important Safety Information: Risk of Thrombotic Adverse Events with Subcutaneous or Inappropriate Intravenous Use of Vivaglobin (Immune Globulin Subcutaneous). (2011). Available from: http://www.fda.gov/BiologicsBloodVaccines/SafetyAvailability/ucm246863.htm

[63] Berger M. l-proline-stabilized human IgG: Privigen^®^ 10% for intravenous use and Hizentra^®^ 20% for subcutaneous use. Immunotherapy (2011) 3(2):163–76. 10.2217/imt.10.108 21322757

[64] Komenda M Stadler D Malinas T Moses M Pragst I Herzog E Assessment of the ability of the Privigen^®^ purification process to deplete thrombogenic factor XIa from plasma. Vox Sang (2014) 107(1):26–36. 10.1111/vox.12119 24329163

[65] Farrugia A Penrod J Bult JM. The ethics of paid plasma donation: a plea for patient centeredness. HEC Forum (2014). 10.1007/s10730-014-9253-5 25234254

[66] Ramírez E Romero-Garrido JA López-Granados E Borobia AM Pérez T Medrano N Symptomatic thromboembolic events in patients treated with intravenous-immunoglobulins: results from a retrospective cohort study. Thromb Res (2014) 133(6):1045–51. 10.1016/j.thromres.2014.03.046 24731561

[67] Daw Z Padmore R Neurath D Cober N Tokessy M Desjardins D Hemolytic transfusion reactions after administration of intravenous immune (gamma) globulin: a case series analysis. Transfusion (2008) 48:1598–601. 10.1111/j.1537-2995.2008.01721.x 18466176

[68] Funk M Lohmann A Keller-Stanislawski B Erhöhte Melderate von schweren hämolytischen Reaktionen nach der intravenösen Gabe von Immunglobulinen. Bulletin zur Arzneimittelsicherheit (2012) 2:15–7.

[69] Food and Drug Administration. Strategies to Address Hemolytic Complications of Immune Globulin Infusions. (2014). Available from: http://www.fda.gov/biologicsbloodvaccines/newsevents/workshopsmeetingsconferences/ucm378388.htm.

[70] Funk M Keller-Stanislawski B Hemolytic Complications of Immune Globulin Infusions – European experience. Presented at the Public Workshop: Strategies to Address Hemolytic Complications of Immune Globulin Infusions. (2014). p. 20–7 Available from: http://www.fda.gov/downloads/BiologicsBloodVaccines/NewsEvents/WorkshopsMeetingsConferences/UCM387078.pdf

[71] Bellac CL Polatti D Hottiger T Girard P Sänger M Gilgen M. Anti-A and anti-B haemagglutinin levels in intravenous immunoglobulins: are they on the rise? A comparison of four different analysis methods and six products. Biologicals (2014) 42(1):57–64. 10.1016/j.biologicals.2013.10.004 24325871

[72] CSL Behring. Health Canada Endorsed Important Safety Information on Privigen^®^, Immune Globulin Intravenous (Human). (2012). Available from: https://www.blood.ca/CentreApps/Internet/UW_V502_MainEngine.nsf/resources/CustomerLetters2012/$file/Privigen-NtoH-Clean_2012-05-24.pdf

[73] Romberg V Effects of the Manufacturing Process on Anti-A titers in IVIG. Presented at the Public Workshop: Strategies to Address Hemolytic Complications of Immune Globulin Infusions. (2014). p. 39–47 Available from: http://www.fda.gov/downloads/BiologicsBloodVaccines/NewsEvents/WorkshopsMeetingsConferences/UCM387079.pdf

[74] Nardini C Anti-A and anti-B haemagglutinin trend analysis during manufacturing process of IVIG. Presented at the Public Workshop: Strategies to Address Hemolytic Complications of Immune Globulin Infusions. (2014). p. 56–66 Available from: http://www.fda.gov/downloads/BiologicsBloodVaccines/NewsEvents/WorkshopsMeetingsConferences/UCM387079.pdf

[75] Hoefferer L Process Modifications to Reduce the Isoagglutinin Levels in Immunoglobulin Products. Presented at the Public Workshop: Strategies to Address Hemolytic Complications of Immune Globulin Infusions. p. 67–78 Available from: http://www.fda.gov/downloads/BiologicsBloodVaccines/NewsEvents/WorkshopsMeetingsConferences/UCM387079.pdf

[76] Siani B Willimann K Wymann S Marques AA Widmer E. Isoagglutinin reduction in human immunoglobulin products by donor screening. Biol Ther (2014) 4(1–2):15–26. 10.1007/s13554-014-0016-2 24841428 PMC4254866

[77] Murphy WG. Disease transmission by blood products: past, present and future. Pathophysiol Haemost Thromb (2002) 32(Suppl 1):1–4. 10.1159/000057291 12214137

[78] Farrugia A. Globalisation and blood safety. Blood Rev (2009) 23(3):123–8. 10.1016/j.blre.2008.10.004 19081166

[79] Oncley J Melin MDA Richert DA Cameron W Gross PM The separation of the antibodies, isoagglutinins, prothrombin, plasminogen and beta1-lipoprotein into subfractions of human plasma. J Am Chem Soc (1949) 71(2):541–50 10.1021/ja01170a048 18112064

[80] Tatford O Keeping ahead in biopharmaceutical manufacturing. Presented to the Bioprocessing Network Annual Conference (2009). Available from: http://www.bioprocessingnetwork.com.au/images/Thursday/Owen_Tatford_bpn09.pdf

[81] Laub R Baurin S Timmerman D Branckaert T Strengers P Specific protein content of pools of plasma for fractionation from different sources: impact of frequency of donations. Vox Sang (2010) 99(3):220–31 10.1111/j.1423-0410.2010.01345.x 20840337 PMC3001115

[82] Stonebraker JS Farrugia A Gathmann B. ESID registry working party, orange JS: modeling primary immunodeficiency disease epidemiology and its treatment to estimate latent therapeutic demand for immunoglobulin. J Clin Immunol (2014) 34(2):233–44. 10.1007/s10875-013-9975-1 24338563

[83] Farrugia A Cassar J Is self-sufficiency in haemotherapies a practical or necessary goal? Blood Transfus (2013) 11(2):183–92. 23245724 10.2450/2012.0148-12 PMC3626469

[84] Australian National Blood Authority. National Report on the Issue and Use of Intravenous Immunoglobulin (IVIg) 2012-13. Available from: http://www.blood.gov.au/sites/default/files/nba-ivig-annual-report-2012-13.pdf

[85] Quinti I Soresina A Guerra A Rondelli R Spadaro G Agostini C IPINet investigators: effectiveness of immunoglobulin replacement therapy on clinical outcome in patients with primary antibody deficiencies: results from a multicenter prospective cohort study. J Clin Immunol (2011) 31(3):315–22. 10.1007/s10875-011-9511-0 21365217

[86] Personalized Medicine Coalition. The Case for Personalized Medicine 2014. (2014). Available from: http://www.personalizedmedicinecoalition.org/Userfiles/PMC-Corporate/file/pmc_the_case_for_personalized_medicine.pdf

[87] Milito C Pulvirenti F Pesce AM Digiulio MA Pandolfi F Visentini M Adequate patient’s outcome achieved with short immunoglobulin replacement intervals in severe antibody deficiencies. J Clin Immunol (2014) 34(7):813–9. 10.1007/s10875-014-0081-9 25047154 PMC4165867

[88] Schwab I Nimmerjahn F. Intravenous immunoglobulin therapy: how does IgG modulate the immune system? Nat Rev Immunol (2013) 13(3):176–89. 10.1038/nri3401 23411799

[89] Czajkowsky DM Hu J Shao Z Pleass RJ. Fc-fusion proteins: new developments and future perspectives. EMBO Mol Med (2012) 4(10):1015–28. 10.1002/emmm.201201379 22837174 PMC3491832

[90] Cashin-Garbutt A Generating an Alternative to IVIG Therapy: An Interview with Professor Richard Pleass, LSTM. (2013). Available from: http://immunologynews.blogspot.com.au/2013/11/generating-alternative-to-ivig-therapy.html

